# Hereditary epidermolysis bullosa: clinical-epidemiological profile of 278 patients at a tertiary hospital in São Paulo, Brazil^☆^

**DOI:** 10.1016/j.abd.2023.06.009

**Published:** 2024-02-24

**Authors:** Chan I. Thien, Vanessa Rolim Bessa, Isadora Zago Miotto, Luciana Paula Samorano, Maria Cecília Rivitti-Machado, Zilda Najjar Prado de Oliveira

**Affiliations:** Department of Dermatology, Hospital das Clínicas, Faculty of Medicine, Universidade de São Paulo, São Paulo, SP, Brazil

**Keywords:** Brazil, Epidemiology, Epidermolysis bullosa, Epidermolysis bullosa, junctional, Epidermolysis bullosa dystrophica, Epidermolysis bullosa simplex, Tertiary healthcare

## Abstract

**Background:**

Epidermolysis bullosa (EB) is a group of rare hereditary diseases, characterized by fragility of the skin and mucous membranes. Epidemiological data on EB in Brazil are scarce.

**Objectives:**

To describe epidemiological aspects of patients with EB diagnosed in the Dermatology Department of a tertiary hospital, from 2000 to 2022.

**Methods:**

An observational and retrospective study was conducted through the analysis of medical records. The evaluated data included clinical form, sex, family history, consanguinity, age at diagnosis, current age, time of follow-up, comorbidities, histopathology and immunomapping, presence of EB nevi and squamous cell carcinomas (SCC), cause of and age at death.

**Results:**

Of 309 patients with hereditary EB, 278 were included. The most common type was dystrophic EB (DEB), with 73% (28.4% dominant DEB, 31.7% recessive DEB and 12.9% pruriginous DEB). Other types were junctional EB with 9.4%, EB simplex with 16.5% and Kindler EB with 1.1%. Women accounted for 53% and men for 47% of cases. Family history was found in 35% and consanguinity in 11%. The mean age at diagnosis was 10.8 years and the current age was 26 years. The mean time of follow-up was nine years. Esophageal stenosis affected 14%, dental alterations affected 36%, malnutrition 13% and anemia 29%. During diagnostic investigation, 72.6% underwent histopathological examination and 92% underwent immunomapping. EB nevi were identified in 17%. Nine patients had SCC. Eleven patients died.

**Study limitations:**

Insufficient data included to medical records, loss to follow-up, and unavailability of genetic testing.

**Conclusions:**

In this study, dystrophic EB predominated and the need for multidisciplinary care for comorbidities and complications was highlighted.

## Introduction

Epidermolysis bullosa (EB) is a group of rare, to date incurable, genetically determined hereditary diseases characterized by fragility of the skin and mucous membranes. Mucocutaneous bullae, erosions and ulcerations occur due to minor trauma, which impacts the quality of life of affected individuals.^1^

The 2020 consensus for the classification of EB^1^ separated the classic forms of EB from other diseases that cause skin fragility, in which the skin cleavage is very superficial (suprabasal), such as peeling skin syndrome.^1^ The clinical presentation is heterogeneous in classic forms of EB. Based on level of skin cleavage, EB is divided into four major types: EB simplex (EBS), junctional EB (JEB), dystrophic EB (DEB), and Kindler EB (KEB).^1^ More than 30 clinical subtypes are recognized and pathogenic mutations have been described in 21 distinct genes, which encode proteins involved in cell adhesion and integrity.^2^

Epidemiological data on EB in Brazil are scarce.^3^, ^4^ The DEBRA Brasil association has a record of 1027 patients in the country; however, this information has not been published. Brazilian studies published so far comprise case reports and case series with clinical characterization and some include genetic analysis.^3^, ^4^, ^5^, ^6^, ^7^, ^8^, ^9^, ^10^, ^11^, ^12^, ^13^

Reliable epidemiological data based on well-characterized cohorts are essential in rare diseases.^14^ They emphasize the need of knowledging the care of these patients and related costs for the health system. They are also important for the design and development of clinical trials and to estimate the number of patients who could benefit from a given treatment.^15^ Phase 3 clinical studies with beremagene geperpavec (B-VEC) in patients with DEB have shown promising results regarding wound healing. This is a topical gene therapy associated with gene editing, with COL7A1 sequences encapsulated in herpes simplex type 1 viral vectors.^16^

The aim of this study was to describe the clinical and epidemiological findings of hereditary EB cases from a tertiary hospital in São Paulo, Brazil.

## Methods

An observational, retrospective, and cross-sectional study was carried out, after approval by the Institutional Ethical Committee, through the analysis of physical and electronic medical records of 309 patients diagnosed with EB, in the Dermatology Division of Hospital das Clínicas, Faculty of Medicine, Universidade de São Paulo (HCFMUSP), Brazil, from January 1, 2000 to December 31, 2022.

The diagnosis was based on the clinical presentation, family history, comorbidities and histopathological and immunomapping exams obtained through skin biopsy material. The diagnoses were grouped into the following categories: EBS, JEB, dominant DEB (DDEB), recessive DEB (RDEB), pruriginous DEB, and KEB. Patients with incomplete data or unavailable medical records were excluded, as were those with suprabasal cleavage diseases (2020 consensus).^1^ Individuals with the disease and family members who were also affected and who were not registered in the service were not actively recruited.

The database used in this study included the clinical form of EB, sex, family history of EB, presence of consanguinity, age at diagnosis, current age, time of follow-up, comorbidities, histopathology, immunomapping, presence of EB nevi and of skin tumors, cause of and age at death (if applicable). Genetic testing was not included, as it is not yet routinely available in the service. Statistical data were presented as percentages and means.

## Results

**Figure 1:**
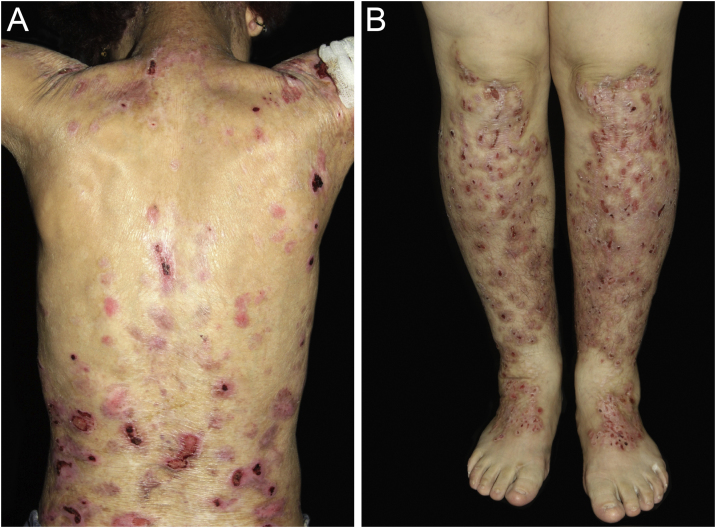
Dominant dystrophic epidermolysis bullosa (DDEB). (A) Generalized form affecting the back. Healing of bullae leads to scarring. (B) Excoriated papules on the lower limbs in pruriginous DEB.

**Figure 2:**
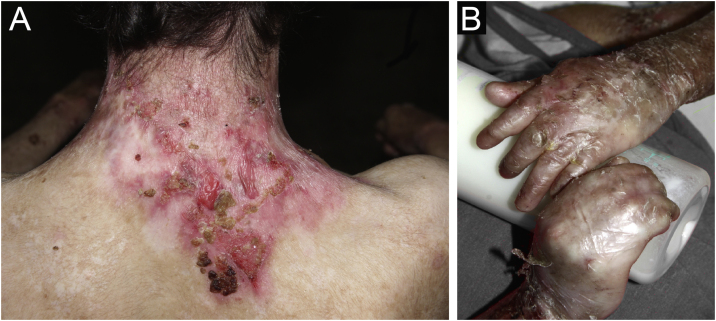
Recessive dystrophic epidermolysis bullosa (RDEB). (A) Bullae and ulcerated areas cause scarring and fibrosis. (B) Pseudosyndactyly of the hands, with fusion digits.

**Figure 3:**
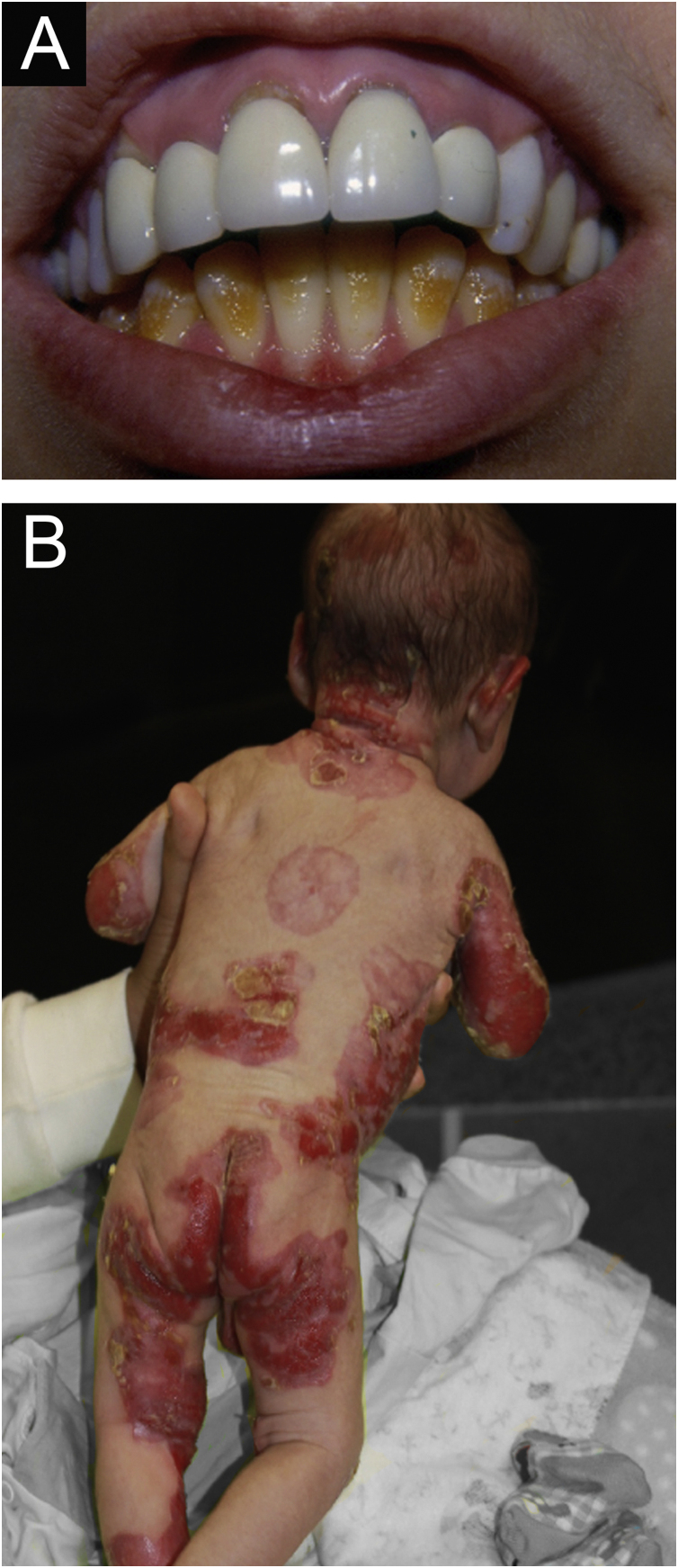
Junctional epidermolysis bullosa (JEB). (A) Dental enamel defects in the lower dental arch, with punctate depressions and yellowish color. In the upper dental arch it is possible to see laminated veneers (dental contact lenses). (B) Baby with skin fragility and denuded areas; other areas with granulation tissue and crusts.

**Figure 4:**
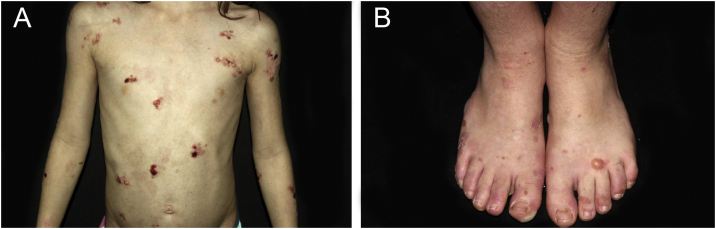
Epidermolysis bullosa simplex (EBS). (A) Child with tense blisters, ulcerations and hematic crusts. Observe the involvement of friction sites (periaxillary region). (B) Healing bullae and erosions on the feet in the localized form of EBS.

**Figure 5:**
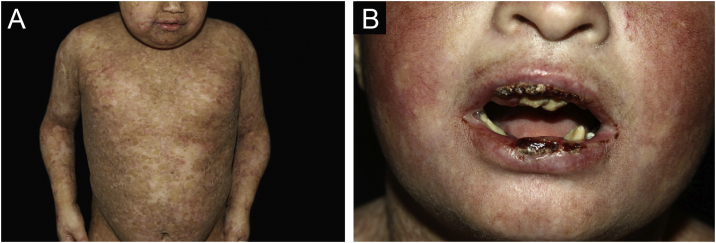
Kindler epidermolysis bullosa (KEB). (A) Scarring lesions with mottled, hypo- and hyperpigmented macules in a child with KEB. (B) Poikiloderma on the face. Crusts and ulcerations on the lips. Teeth in poor condition.

Of the 309 patients with EB, 31 were excluded due to incomplete data. Of the 278 included patients, 147 (53%) were women and 131 (47%) were men. The most common type was the dystrophic form, with 203 patients (73%), of which 79 (28.4%) were DDEB (Fig. 1A), 36 (12.9%) were pruriginous DEB (Fig. 1B) and 88 (31.7%) were RDEB (Fig. 2A and B). Other types were JEB (Fig. 3A and B) with 26 (9.4%) patients, EBS (Fig. 4A and B) with 46 (16.5%), and Kindler EB (Fig. 5A and B) in three (1.1%). A positive family history was found in 96 (35%) patients and consanguinity was reported by 31 (11%) patients. The distribution of patients by type of EB in relation to sex, family history, and consanguinity is shown in Table 1.

**Table 1:** Distribution of the type of epidermolysis bullosa in relation to sex, family history and consanguinity.

Sex				Family history				Consanguinity		
Type	Male	Female	Total	Yes	No	Not informed	Total	Yes	No	Total
EBS	23	23	46	14	24	8	46	5	41	46
JEB	12	14	26	10	10	6	26	3	23	26
DDEB	37	42	79	31	40	8	79	10	69	79
RDEB	44	44	88	23	50	15	88	8	80	88
Pruriginous DEB	13	23	36	17	12	7	36	5	31	36
KEB	2	1	3	1	2	0	3	0	3	3
	131	147	278	96	138	44	278	31	247	278
	47%	53%		35%	50%	16%		11%	89%	

The mean age at the diagnosis was 10.8 years (ranging from one day of life to 82 years, as detailed in Table 2). Ninety-three (33.5%) patients were diagnosed at less than one year of life, while 103 (37%) were diagnosed between one and ten years old. Twenty-nine (10%) patients were diagnosed between 11 and 20 years and 52 (19%) patients were diagnosed with EB between 21 and 80 years, while one male patient was diagnosed at 82 years old.

**Table 2:** Distribution of the type of epidermolysis bullosa according to age at diagnosis and current age. In the case of patients who died, the age at death was considered.

Age at diagnosis												
	0 to 1 year	1 to 10	11 to 20	21 to 30	31 to 40	41 to 50	51 to 60	61 to 70	71 to 80	81 to 90	Total	
EBS	20	16	7	2	1	0	0	0	0	0	46	16.5%
JEB	11	9	1	0	1	2	2	0	0	0	26	9.4%
DDEB	24	32	8	3	8	3	0	1	0	0	79	28.4%
RDEB	38	40	6	1	2	0	1	0	0	0	88	31.7%
Pruriginous DEB	0	5	5	5	7	7	2	4	0	1	36	12.9%
KEB	0	1	2	0	0	0	0	0	0	0	3	1.1%
	93	103	29	11	19	12	5	5	0	1	278	

Current age (or at death)											
	0 to 10	11 to 20	21 to 30	31 to 40	41 to 50	51 to 60	61 to 70	71 to 80	81 to 90	Total	
EBS	8	14	15	5	3	1	0	0	0	46	16.5%
JEB	6	6	9	0	0	1	3	1	0	26	9.4%
DDEB	13	28	15	8	4	7	3	0	1	79	28.4%
RDEB	13	41	25	6	0	2	1	0	0	88	31.7%
Pruriginous DEB	0	2	4	9	6	5	3	5	2	36	12.9%
KEB	0	1	2	0	0	0	0	0	0	3	1.1%
	40	92	70	28	13	16	10	6	3	278	

Considering the current age (Table 2), the mean was 26 years old. For the calculation, the age considered in the case of patients who died was the age at death. Only 40 (14%) individuals were younger than ten years old, while 92 (33%) patients were aged between 11 and 20 years. In the age group between 21 and 80, there were 143 (51%) patients, while three were over 81 years old.

The mean time of follow-up was nine years, excluding six patients who only attended for histopathological and immunomapping exams and did not return for follow-up at the outpatient clinic. For deceased patients, the date of the last appointment before death was considered.

Regarding extracutaneous involvement (Table 3), 40 (14%) patients had different degrees of dysphagia (esophageal stenosis). Of these, 25 (62.5%) were RDEB, ten (25%) were DDEB, two (5%) were pruriginous DEB, two (5%) were JEB, one (2.5%) was EBS and none was KEB.

**Table 3:** Distribution of the main comorbidities in the different types of epidermolysis bullosa.

Comorbidities						
	Esophageal stenosis	Dental alteration	Malnutrition	Anemia	Blood transfusion	Orthopedic alteration
EBS	1	13	3	5	0	2
JEB	2	3	1	4	0	2
DDEB	10	28	6	22	0	15
RDEB	25	54	24	47	6	47
Pruriginous DEB	2	0	0	2	0	0
KEB	0	2	1	1	0	1
N of affected patients	40	100	35	81	6	67
% (n of affected/total patients)	14%	36%	13%	29%	2%	24%

Dental alterations were identified in 100 (36%) patients, among which 54 (54%) were RDEB, 28 (28%) were DDEB, 13 (13%) were EBS, three (3%) were JEB, two (2 %) were KEB, whereas none was pruriginous DEB.

Malnutrition was assessed using body mass index (BMI ≤ 18) and affected 35 (13%) individuals. Of these, 24 (68.6%) were RDEB, six (17.1%) were DDEB, three (8.5%) were EBS, one (2.9%) was JEB and one (2.9%) was KEB.

Anemia was a common finding, with 81 (29%) affected patients, of which six (2%) underwent blood transfusions, all of which were RDEB.

Orthopedic involvement was observed in 67 (24%) patients and those with pseudosyndactyly in the hands (59 patients) were referred to the orthopedics outpatient clinic.

**Figure 6:**
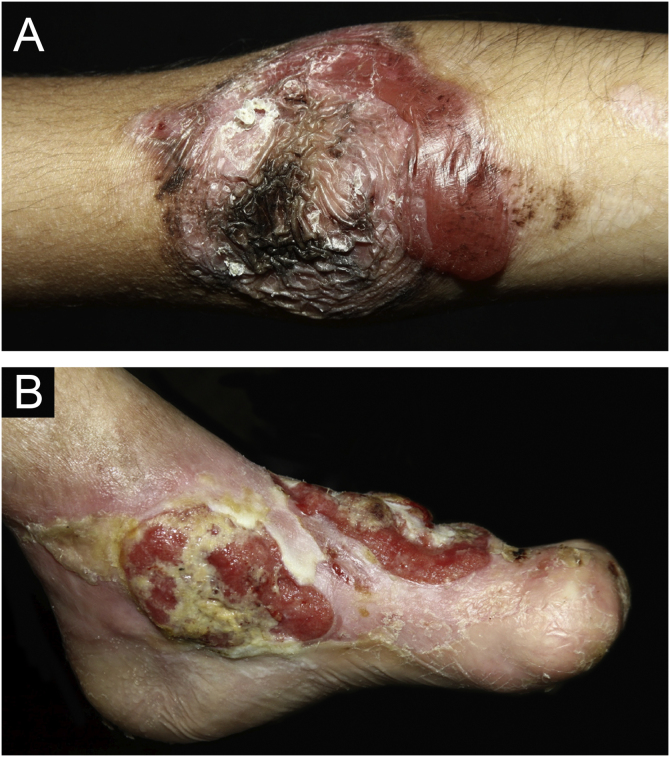
(A) Epidermolysis bullosa nevus (EBN) on the elbow, in a patient with RDEB. (B) Extensive area with ulceration and exophytic lesions, with evolution to squamous cell carcinoma (SCC) on the foot.

Epidermolysis bullosa nevi (EB nevi) were identified in 47 (17%) individuals, with a total of 90 nevi, since the same patient had one to five lesions (Fig. 6A). Ten patients were DDEB, 29 were RDEB, two were JEB, five were EBS, and one was pruriginous DEB.

Pyloric atresia was identified in an EBS patient with severe cutaneous involvement. Muscular dystrophy was observed in one EBS patient. Dilated cardiomyopathy was identified in a 30-year-old KEB patient who was lost to follow-up and in an RDEB patient who died at 20 years old.

Dermatological findings unrelated to EB included seborrheic dermatitis (1), atopic dermatitis (1), and pityriasis amiantacea (1). Folliculitis decalvans was identified in two individuals, both with dystrophic EB. Cicatricial alopecia affected two JEB patients. Table 4 shows other extracutaneous diseases found in patients in this cohort.

**Table 4:** Other extracutaneous manifestations identified in patients in this study, with the number of affected individuals in parentheses.

Extracutaneous manifestations		
**Ophthalmological**		
Glaucoma (3)	Amaurosis (3)	Leukocoria (1)
Corneal opacity (5)	Keratitis/Keratopathy (2)	Strabismus (1)
Corneal Ulcer (2)	Conjunctivitis (1)	Symblepharon (1)
Blepharitis (1)	Retinal detachment (1)	Myopia (2)
Macula staphyloma (1)		
**Gastroenterological**		
Esophageal stenosis (40)	Megacolon (1)	Dyspeptic Syndrome (3)
Gastroesophageal reflux disease (3)	Constipation (7)	
**Otorhinolaryngological**		
Otitis (3)	Hoarseness (2)	Pharyngeal Dyskinesia (1)
Hypoacusis (1)	Laryngomalacia (1)	Right Maxillary Sinus Tumor (1)
Obstructive sleep apnea and hypopnea Syndrome (1)		
**Orthopedic**		
Pseudosyndactyly (59)	Bone malformation (2)	Osteomyelitis (1)
Joint deformity (1)	Lower limb asymmetry (1)	
**Endocrinological**		
Hypothyroidism (8)	Diabetes mellitus (5)	Short stature (2)
Osteoporosis (3)	Delayed puberty (1)	Precocious puberty (1)
Insulin-dependent diabetes mellitus (1)	Thyroid nodule (1)	
**Hematological**		
Anemia (81)	Sickle cell trait (2)	G6PD deficiency (1)
Multiple myeloma (1)	Idiopathic thrombocytopenic purpura (1)	
**Psychiatric**		
Depression (1)	Suicidal ideation (1)	Panic syndrome (1)
**Pneumological**		
Asthma (3)	Allergic rhinitis (2)	Tracheal stenosis (1)
**Cardiological**		
Arterial hypertension (13)	Dilated cardiomyopathy (2)	Chagas disease (1)
Angina (1)	Heart disease (ASD, aortic and pulmonary stenosis) (1)	
**Urological**		
Urinary infection (3)	Cryptorchidism (2)	Urethral stenosis (1)
**Nephrological**		
Nephropathy (1)	IgA nephritis (1)	

During the diagnostic investigation process, 202 (72.6%) individuals underwent a total of 302 biopsies for histopathology. Immunomapping was performed in 256 (92%) patients, with a total of 359 exams performed during this period. Nine underwent biopsy only, 63 underwent immunomapping only, 193 patients underwent both tests and 13 underwent neither test. The classification of the EB type of the 13 patients who did not undergo any examination took into account family history and compatible clinical aspects, although the diagnosis was not confirmed by the laboratory. These patients did not undergo skin biopsy or immunomapping for social reasons (unavailability to return to the hospital) or lack of consent from their guardians or the patients themselves (five newborns, five children under 12 years old, and three adults, aged 17, 27 and 49 years).

During the follow-up period, nine (3.2%) patients were diagnosed with 11 squamous cell carcinomas (SCC). One JEB patient had three SCCs (one in each lower limb and one on the back). The location of SCC (Fig. 6B) in the remaining patients was in the lower limbs in six (one JEB, four RDEB, and one DDEB) and back in one patient (RDEB). In one of the RDEB patients, the location of the SCC is unknown due to lack of information in the medical records. Transtibial amputation was performed in three patients (two males with RDEB at 18 and 19 years old and one female with DDEB at 27 years old). Five (55.5%) had metastasis and died, including three cases of RDEB (the age at death of two patients was 18 years old and one was 20 years old), one DDEB (28 years old), and one JEB (57 years old).

In total, 11 patients died, five RDEB (18, 18, 20, 22 and 24 years old), five DDEB (one, 11, 15, 15, 28 years old), and one JEB (57 years old). The cause of death for five of these patients was complications from metastatic SCC. Three died due to sepsis caused by bronchopneumonia (two DDEB, both aged 15 years, and one RDEB aged 22). A 24-year-old patient with RDEB had broncho-aspiration as the cause of death. Two children with DDEB, aged one year and 11 years, died of unknown causes.

Patients who did not attend consultations in dermatology or other specialties at HCFMUSP for more than two years were considered lost to follow-up. In total, 158 (57%) patients had a last appointment date before 12/31/2020. Three patients died between 2021 and 2022.

## Discussion

In the present study of hereditary EB, sex predilection was not observed and the number of women and men affected was similar, as previously described in the literature.^2^, ^17^ Dystrophic EB was the most common type (73%), at a similar proportion to that found in a study carried out in Iran (75.7%).^17^ Studies from other countries described a lower frequency of DEB, such as Canada (35%),^18^ Australia (35%),^19^ the Netherlands^20^ with 34.7% (DDEB 23.3% and RDEB 11.4%), Germany (32.5%)^21^ and India (17%).^22^ The predominance of more severe forms of EB in the present study is due, in part, to difficulties in having access to specialized medical care,^23^ the underdiagnosis of more localized forms of EBS, and the fact that HCFMUSP is a tertiary hospital, a national reference in the care of patients with EB, and in dealing with more complex cases.

In the present study, JEB affected 9.4% of patients, while KEB affected 1.1% and EBS 16.5. The frequency of JEB in studies from Iran,^17^ Canada^18^ and Australia^19^ was 11%; whereas it was 18.8% in the Netherlands^20^ and 17.1% in Germany.^21^ As for KEB, 3% was found in Iran,^17^ 0.3% in Australia,^19^ 0.9% in the Netherlands^20^ and 0.5% in Germany.^21^ The percentages found in the present study were similar in both types of EB. In relation to EBS, the frequency found is lower than that described in the literature, for the previously mentioned reason that the studied population comes from a tertiary hospital. According to a 2021 epidemiological study from the Netherlands,^20^ EBS was diagnosed in 45.7%. In this Dutch cohort, 90.5% of the patients had a genetically confirmed diagnosis.^20^ Epidemiological data from 1779 patients from Germany, published in 2022,^21^ showed 39.3% (700) of EBS patients. This study included patients from tertiary hospitals, dermatology outpatient clinics, laboratories that performed the diagnosis, and patient organizations.

The mean current age found in the present study was 26 years old and only 40 (14%) individuals were under ten years old. The high number of patients at pubertal age (over 11 years old), allows the authors to infer an increase in life expectancy and reflects the improvement in multidisciplinary care, mainly in relation to nutrition, infection, and pain management.

Regarding consanguinity, only 31 patients (11%) reported its presence and DDEB was the most common type in the study population (32,2%). As most patients did not undergo genetic testing, the diagnosis was not confirmed by such a method. The literature has shown that the number of patients with an autosomal recessive form of EB may be associated with communities with high rates of parental consanguinity.^24^

The most common patient complaints were dental alterations (36%), followed by anemia (29%) and orthopedic diseases (24%). In the Iranian study, the most common complaint was dysphagia, followed by dental alterations.^17^ These dental findings in JEB are due to tooth enamel hypoplasia and the presence of punctate depressions (Fig. 3A), which are pathognomonic clinical findings in this type of EB. These changes lead to early loss of teeth and the presence of cavities. In RDEB, microstomia and ankyloglossia are observed and make daily dental hygiene a difficult task.^25^ Poor oral hygiene care must always be taken into account in most patients with EB (Fig. 5B).

Pseudosyndactyly of the hands (Fig. 2B) and feet is a musculoskeletal complication that occurs mainly in RDEB.^25^ The use of gloves or bandages to keep the fingers separated is advised in patients with dystrophic EB. In 2011, the orthopedic group specialized in hands at HCFMUSP, in colaboration with the Pediatric Dermatology team, published data on 59 patients with EB and hand deformities, of which 25 underwent surgery, with a high rate of reoperation.^26^ A 2019 study carried out in China^27^ reported 11 RDEB patients who were submitted to surgery and were followed for two years, 9 of which showed loss of hand functionality due to the re-narrowing of the interdigital spaces, with digit adhesion and flexion of the metacarpophalangeal and interphalangeal joints. The others two patients were reoperated after one year due to recurrence.

The United States National EB Registry (NEBR), which has a sample of 3280 patients followed for a period of 16 years (1986‒2002)^28^, ^29^ remains the largest epidemiological study on EB. It demonstrated that the frequency of esophageal stenosis is 79.1% in patients with severe RDEB, 37.2% for intermediate RDEB, and 14.3% for JEB, with lower rates in other EB subtypes.^28^ In the present study, esophageal stenosis was identified in 14% of the total number of individuals, with 28.4% and 7.7% being the frequencies observed in patients with RDEB and JEB, respectively.

In 2005, a study was published by the Gastroenterology and Pediatric Dermatology Services at HCFMUSP,^30^ which reported 19 patients with severe forms of EB (DEB and JEB) treated between January 1999 and April 2001. Vomiting and dysphagia were reported in 16 (84.2%) cases, choking by 14 (73.7%) and intestinal constipation by 14 (73.7%) patients. Anemia affected all cases. Regarding nutritional aspects, 12 (63.1%) had weight and height below the 2.5^th^ percentile.^30^ In the present study, malnutrition affected 35 patients (12%). The lowest percentage found herein is associated with the methodology of this study, which included all forms of EB.

EB nevus is described as an acquired, eruptive, asymmetric nevus and affects around 14% of EB patients, in its different clinical forms.^31^ EB nevus generally occurs at sites of previous bullae and may spontaneously regress. However, in theory, melanoma could arise from an EB nevus or from pigmented lesions that clinically mimic the EB nevus. Therefore, any morphological change in an EB nevus, especially the appearance of nodules or ulceration, indicates the need for histopathological study.^32^ In the present study, EB nevi were identified in 17% of the patients. In 2014, the HCFMUSP Pediatric Dermatology team described 13 DEB patients with EB nevi, five of which were biopsied due to the atypical dermoscopic characteristics of the nevi. Two corresponded to atypical nevus or atypical lentiginous proliferation on histopathology.^7^ In 2005, a group from Austria^33^ described dermoscopic features of 23 EB nevi from 11 patients, with criteria usually associated with melanoma such as multicomponent pattern (20 of 23), atypical pigmented network (17 of 23), irregular globules and spots (16 of 23), irregular pigmentation (22 of 23) and atypical vascular pattern (7 of 23). The same group indicated histological evaluation of highly suspicious lesions.

Pyloric atresia associated with EB was found in a 28-year-old female patient with EBS and extensive cutaneous involvement. It is a distinct subtype, which generally occurs in severe EB phenotypes.^34^ When it occurs concomitantly with severe EBS, it is associated with a mutation in the Plectin (PLEC) gene, a hemidesmosome-forming protein.^2^ The genetic diagnosis of this specific patient has not yet been carried out.

A 22-year-old male EBS patient with a genetic diagnosis of a mutation in the PLEC gene had muscular dystrophy, as previously mentioned in the literature. It may be present at birth or appear in the first days of life in children with severe skin disease,^35^ or it may establish insidiously in late childhood or adulthood.^25^ In a 2021 Dutch study,^20^ two patients with EBS and muscular dystrophy died at ages 43.7 and 46 due to heart failure.

A higher risk of dilated cardiomyopathy is seen in patients with severe RDEB, with a cumulative risk of 4.51% by 20 years old.^36^ Among the patients included in this study, cardiomyopathy affected one RDEB patient, who died with 20 years, and one KEB patient with 30 years old, who was lost to follow-up.

**Figure 7:**
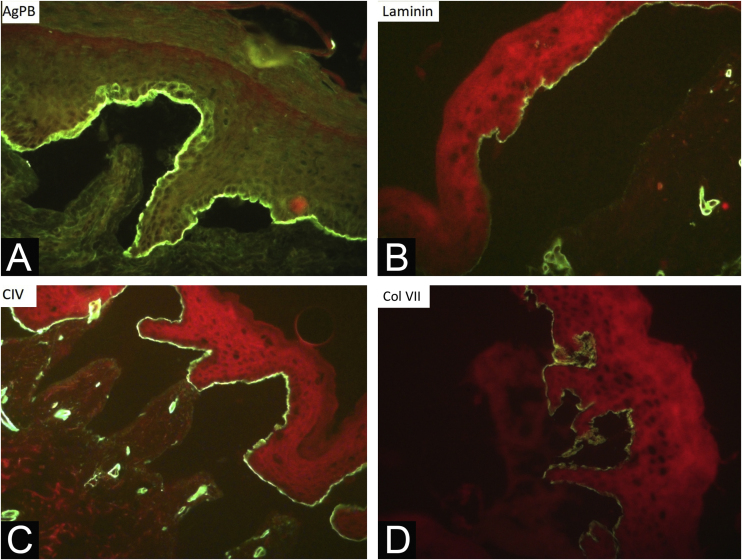
Immunomapping of dominant dystrophic epidermolysis bullosa (DDEB). Observe cleavage in the sublamina densa, with marker deposition and fluorescence on the roof of the bullae. The markers used are: (A) Bullous pemphigoid antigen, (B) laminin, (C) collagen IV and (D) collagen VII.

During the assessed period, 302 skin biopsies and 359 immunomappings were performed. Some tests were repeated due to inconclusive reports, in biopsied bullae that were undergoing the process of re-epithelialization or when cleavage was absent. The histological examination of the skin biopsy must be performed on a recent intact bulla (affected skin). Cleavage is intraepidermal in cases of EBS and subepidermal in JEB and DEB, and it is not possible to differentiate these two forms through routine histopathology.^10^ The cleavage plane is variable in KEB. Immunomapping can be carried out on a sample of healthy skin with an induced vesicle or on a recent and ideally small vesicle so that it does not rupture during biopsy. This is an immunofluorescence technique and allows the observation of the cleavage plane, making it possible to differentiate among simple, junctional, and dystrophic forms.^10^ The markers used in the Department of Dermatology at HCFMUSP are bullous pemphigoid antigen (hemidesmosome marker), anti-laminin 5 antibody (lamina lucida), anti-collagen IV antibody (lamina densa) and anti-collagen VII antibody (sublamina densa and anchoring fibrils). In EBS, cleavage occurs in the basal layer. In JEB, the level of cleavage occurs in the lamina lucida, while in DEB it occurs in the sublamina densa (Fig. 7).^10^ In RDEB, there is an absence or marked decrease in fluorescence with collagen VII. In 2021, a study from India^37^ showed the usefulness of immunomapping as a diagnostic method, given the unavailability of electron microscopy and genetic tests. The diagnosis of 104 EBS, 28 JEB, and 26 DEB was performed with a concordance rate of 41.3%, with higher rates in EBS cases.

Researching mutations in genes associated with EB is the reference technique for defining the EB subtype and would prevent the need for biopsy and immunomapping. It can be performed more quickly and at lower costs, with higher mutation detection rates, due to advances in sequencing technology.^38^ In HCFMUSP, genetic testing has been carried out as part of the research, but it is not yet widely available in outpatient practice, as the exam is not covered by the Brazilian Unified Health System (SUS, *Sistema Único de Saúde*).

In some forms of EB, especially RDEB, there is an increased risk of SCC in late adolescence and early adulthood. A recent study by a group from Australia and New Zealand^39^ showed 16 RDEB patients with 161 primary SCCs, with an average of 10 SCCs per individual. Of these tumors, 70% appeared on the hands and feet, mainly in areas of chronic ulcers and wounds that did not heal. Metastasis was detected in 11 (68.7%) of these patients. Partial limb amputation was performed in seven cases.^39^ Of the 9 patients diagnosed with SCC in the present study, seven (77.7%) were located in the lower limbs and five (55.5%) presented metastasis. Only one individual with JEB had more than one SCC (three tumors), while three patients underwent transtibial amputation. SCC is the main cause of death in this group of patients and the cumulative risk of death from metastasis is 38.7% at 35 years of age and 78.8% at 55 years of age. Most patients with RDEB die from metastatic SCC within five years of the diagnosis.^29^

The low number of deaths (11) identified in the present study may be related to the multidisciplinary care provided to the patients. However, there is an information bias, as these data are only recorded in the medical file when the death occurs in a hospital in the HCFMUSP complex. Information on deaths occurring at home or in other hospitals is not available due to the lack of a unified national database. Five RDEB patients died at a mean age of 20.4 years, while the mean age at death of five DDEB patients was 14 years. A recent study in the Netherlands showed that the mean age of death was 32.5 years in DEB, while in RDEB was 28.2 years and in DDEB was 33.3 years.^20^

The main limitations of the present study were that is retrospective, with a long evaluation period (2000-2022), with lack of information in some medical records and loss of follow-up of some patients. Another point is the unavailability of routine genetic tests for patients with EB in the service and in the public health system, which can make it difficult to classify the clinical form of EB. Although this is not a multicentric study, it represents the first study with epidemiological data on hereditary epidermolysis bullosa in the Brazilian population.

In Brazil, HCFMUSP is one of the few multidisciplinary reference centers specialized in EB. Access to these services is difficult, as it depends on referrals from basic health units, which are often distant and even located in other states, since the service attends patients from all over the country. Another critical problem is the lack of supplies for patients basic needs, such as non-adherent dressings and even regular dressings. In many healthcenters, EB patients are often not adequately diagnosed and treated. Underdiagnosis is common and the few epidemiological data that are published don’t reflect the reality of the incidence and prevalence of rare diseases, such as EB. Similar to what occurs in Canada, there are no centers specialized in the care of adults with EB and they continue to be cared for in pediatric outpatient clinics. Adult life issues, such as sexuality, employment, independence, and skin cancer management are dealt with under restricted conditions, and referral to other specialties is advised.^40^

## Conclusion

In rare diseases such as EB, reliable epidemiological data allow the identification of epidemiological trends.^14^ The present study demonstrates the predominance of more severe forms, probably because it is a tertiary hospital, and highlights the need for multidisciplinary care, especially in relation to comorbidities and complications, such as anemia, dental, orthopedic and gastrointestinal tract diseases, and squamous cell carcinoma. In Brazil, it is necessary to create a database unifying clinical information from the entire population, aiming to identify these trends and allow better targeting of health policies.

## Financial support

The study was funded by *Fundo de Apoio à Dermatologia do Estado de São Paulo* - Sebastião Sampaio (FUNADERSP), PROJECT 105‒2022, provided by the Brazilian Society of Dermatology - Regional do Estado de São Paulo (SBD-RESP) and by the Department of Dermatology, Hospital das Clínicas, Universidade de São Paulo.

## Authors' contributions

Chan I Thien: Design and planning of the study; data collection, or analysis and interpretation of data; statistical analysis; drafting and editing of the manuscript or critical review of important intellectual content; effective participation in the research orientation; intellectual participation in the propaedeutic and/or therapeutic conduct of the studied cases; critical review of the literature; approval of the final version of the manuscript.

Vanessa Rolim Bessa: Data collection, or analysis and interpretation of data; statistical analysis; intellectual participation in the propaedeutic and/or therapeutic conduct of the studied cases; approval of the final version of the manuscript.

Isadora Zago Miotto: Data collection, or analysis and interpretation of data; statistical analysis; intellectual participation in the propaedeutic and/or therapeutic conduct of the studied cases; approval of the final version of the manuscript.

Luciana Paula Samorano: Design and planning of the study; drafting and editing of the manuscript or critical review of important intellectual content; effective participation in research orientation; intellectual participation in the propaedeutic and/or therapeutic conduct of the studied cases; approval of the final version of the manuscript.

Maria Cecília Rivitti-Machado: Design and planning of the study; drafting and editing of the manuscript or critical review of important intellectual content; effective participation in research orientation; intellectual participation in the propaedeutic and/or therapeutic conduct of the studied cases; approval of the final version of the manuscript.

Zilda Najjar Prado de Oliveira: Design and planning of the study; drafting and editing of the manuscript or critical review of important intellectual content; statistical analysis; effective participation in research orientation; intellectual participation in the propaedeutic and/or therapeutic conduct of the studied cases; approval of the final version of the manuscript.

## Conflicts of interest

None declared.
